# ^1^H and ^31^P MR Spectroscopy to Assess Muscle Mitochondrial Dysfunction in Long COVID

**DOI:** 10.1148/radiol.233173

**Published:** 2024-12-24

**Authors:** Lucy E. M. Finnigan, Mark Philip Cassar, Mehrsa Jafarpour, Antonella Sultana, Zakariye Ashkir, Karim Azer, Stefan Neubauer, Damian J. Tyler, Betty Raman, Ladislav Valkovič

**Affiliations:** From the Oxford Centre for Clinical Magnetic Resonance Research (OCMR), Division of Cardiovascular Medicine, Radcliffe Department of Medicine, University of Oxford, John Radcliffe Hospital, Headley Way, Oxford OX3 9DU, UK (L.E.M.F., M.P.C., M.J., A.S., Z.A., S.N., D.J.T., B.R., L.V.); Oncology and Haematology Centre, Churchill Hospital, Oxford, UK (A.S.); Axcella Therapeutics, Cambridge, Mass (K.A.); and Institute of Measurement Science, Slovak Academy of Sciences, Bratislava, Slovakia (L.V.).

## Abstract

**Background:**

Emerging evidence suggests mitochondrial dysfunction may play a role in the fatigue experienced by individuals with post–COVID-19 condition (PCC), commonly called long COVID, which can be assessed using MR spectroscopy.

**Purpose:**

To compare mitochondrial function between participants with fatigue-predominant PCC and healthy control participants using MR spectroscopy, and to investigate the relationship between MR spectroscopic parameters and fatigue using the 11-item Chalder fatigue questionnaire.

**Materials and Methods:**

This prospective, observational, single-center study (June 2021 to January 2024) included participants with PCC who reported moderate to severe fatigue, with normal blood test and echocardiographic results, alongside control participants without fatigue symptoms. MR spectroscopy was performed using a 3-T MRI system, measuring hydrogen 1 (^1^H) and phosphorus 31 (^31^P) during exercise and recovery in the gastrocnemius muscle. General linear models were used to compare the phosphocreatine recovery rate time constant (hereafter, τ_PCr_) and maximum oxidative flux, also known as mitochondrial capacity (hereafter, Q_max_), between groups. Pearson correlations were used to assess the relationship between MR spectroscopic parameters and fatigue scores.

**Results:**

A total of 41 participants with PCC (mean age, 44 years ± 9 [SD]; 23 male) (mean body mass index [BMI], 26 ± 4) and 29 healthy control participants (mean age, 34 years ± 11; 18 male) (mean BMI, 23 ± 3) were included in the study. Participants with PCC showed higher resting phosphocreatine levels (mean difference, 4.10 mmol/L; *P* = .03). Following plantar flexion exercise in situ (3–5 minutes), participants with PCC had a higher τ_PCr_ (92.5 seconds ± 35.3) compared with controls (51.9 seconds ± 31.9) (mean difference, 40.6; 95% CI: 24.3, 56.6; *P* ≤ .001), and Q_max_ was higher in the control group, with a mean difference of 0.16 mmol/L per second (95% CI: 0.07, 0.26; *P* = .008). There was no correlation between MR spectroscopic parameters and fatigue scores (*r* ≤ 0.25 and *P* ≥ .10 for all).

**Conclusion:**

Participants with PCC showed differences in τ_PCr_ and Q_max_ compared with healthy controls, suggesting potential mitochondrial dysfunction. This finding did not correlate with fatigue scores.

© The Author(s) 2024. Published by the Radiological Society of North America under a CC BY 4.0 license.

*Supplemental material is available for this article.*

See also the editorial by Parraga and Eddy in this issue.

## Graphical abstract

**Figure:**
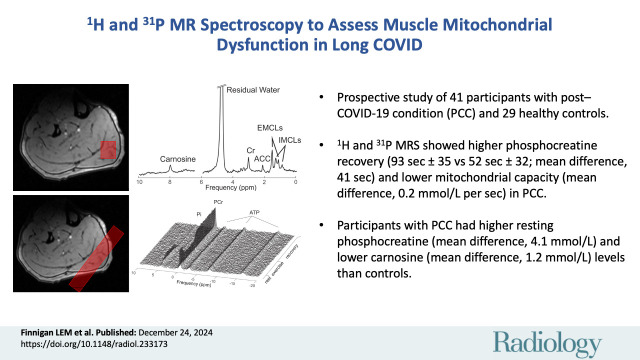

**Summary**

MR spectroscopy showed altered phosphocreatine recovery and mitochondrial capacity in participants with post–COVID-19 condition, also called long COVID, compared with controls, suggesting mitochondrial dysfunction; however, these changes did not correlate with fatigue severity.

**Key Results**

■ In this prospective study of 41 participants with post–COVID-19 condition (PCC) and 29 healthy controls, proton and phosphorus MR spectroscopy revealed a higher phosphocreatine recovery rate time constant (92.5 seconds ± 35.3 vs 51.9 seconds ± 31.9 [*P* < .001]; mean difference, 40.6 seconds [*P* ≤ .001]) and lower mitochondrial capacity (mean difference, 0.16 mmol/L per second; *P* = .008) in participants with PCC.
■ Participants with PCC showed higher resting phosphocreatine (mean difference, 4.10 mmol/L; *P* = .03) and lower carnosine (mean difference, 1.15 mmol/L; *P* = .007) levels compared with controls.
■ No correlations were found between the Chalder fatigue questionnaire (CFQ-11) scores and MR spectroscopic parameters linked to phosphocreatine recovery (r ≤ 0.25 and *P* ≥ .10 for all variables).

## Introduction

The COVID-19 pandemic impacted global health, causing acute illness of varying severity and prolonged symptoms, even after milder infections (1). Post–COVID-19 condition (PCC), commonly called long COVID, refers to these lasting effects (2) that include disabling fatigue, breathlessness, and brain fog (3). Previous studies using tissue biopsies have highlighted the role of mitochondrial dysfunction in promoting fatigue following viral infections (4), potentially explaining the fatigue experienced in individuals with PCC (5). However, there are limited in vivo studies that have noninvasively assessed mitochondrial dysfunction in these patients.

Mitochondria provide 90%–95% of the total energy production of the body, meaning impediments to mitochondrial function can severely affect energy production (6). Mitochondria also play a critical role in the body’s antiviral immune response (7). Early studies investigating SARS-CoV-2 infections observed that the virus can overcome mitochondrial antiviral defenses and impair key mitochondrial processes, such as oxidative phosphorylation, ultimately leading to cell death (8). More recently, it has been suggested that COVID-19 infections may block the transcription of genes encoding key mitochondrial oxidative phosphorylation proteins, which in turn activates glycolysis and an immune stress response (9). Such interference with cellular metabolism and glycolysis upregulation may contribute to the fatigue experienced by individuals with PCC (10).

Exercise studies supported this hypothesis, showing reduced rates of fatty acid oxidation and higher levels of arterial lactate in participants with PCC following graded exercise, consistent with mitochondrial dysfunction (11). Researchers are actively investigating the cause of fatigue symptoms experienced by patients with PCC (12). A recent randomized double-blinded clinical trial found that AXA1125, an endogenous metabolic modulator developed by Axcella Therapeutics, alleviated symptoms of mental and physical fatigue among patients with PCC (13). Post hoc analysis revealed significant improvements in parameters linked to mitochondrial function in those that responded to treatment.

Mitochondrial function can be noninvasively assessed using proton (hydrogen 1 [^1^H]) and phosphorus 31 (^31^P) MR spectroscopy. These well-established techniques enable the quantification of intramyocellular lipid, acetylcarnitine, and carnosine content, which are key components in muscle energy metabolism. Intramyocellular lipids are related to insulin sensitivity, a factor closely related to maximum oxidative flux, also known as mitochondrial capacity (hereafter, Q_max_) (14). Acetylcarnitine fulfils a major role in the translocation of long chain fatty acids from cytosol to the mitochondrial matrix, helping maintain pyruvate dehydrogenase activity (15), also relevant for insulin sensitivity (16). Carnosine is a pH buffer, and its supplementation has been suggested to improve mitochondrial function (17). Exercise may also be used to examine the efficiency of muscle metabolism and indirectly, mitochondrial function. Specifically, the ^31^P MR spectroscopic measurements focus on in vivo quantification of phosphocreatine recovery (ie, phosphocreatine recovery rate time constant [hereafter, τ_PCr_]), pH, inorganic phosphate, and adenosine diphosphate (ADP) during and following an exercise stimulus. From this, mitochondrial function and pH homeostasis can be explored (18).

The aim of this study was to compare mitochondrial function between participants with fatigue-predominant PCC and healthy control participants using in vivo ^1^H and ^31^P MR spectroscopy. The secondary aim was to investigate the relationship between MR spectroscopic parameters and fatigue as assessed using the 11-item Chalder fatigue questionnaire (CFQ-11).

## Materials and Methods

### Study Design

This prospective, observational, single-center study was approved by the Health Research Authority Fast Track and North West Preston Research ethics committees (reference: 21/FT/0158 and 20/NW/0235, respectively). Recruitment occurred from June 2021 to January 2024. All participants provided written informed consent. Data are presented in accordance with STROBE (Strengthening the Reporting of Observational Studies in Epidemiology) guidelines.

### Participants

The study enrolled 41 participants with PCC and 29 healthy control participants. Inclusion criteria for participants with PCC were age 18–65 years with clinically suspected COVID-19 confirmed through a positive polymerase chain reaction test, antibody test, or general practitioner diagnosis at the time of infection. Participants with PCC must not have been hospitalized with noninvasive or invasive ventilatory support during COVID-19 infection and were required to be free from COVID-19 infection for at least 3 months prior to enrollment. Participants were required to have moderate to severe fatigue (ie, Likert scale >16) as assessed using the CFQ-11 (19), taking into account pre–COVID-19 fatigue status. Exclusion criteria included medical conditions that could contribute to fatigue symptoms, such as severe anemia, hypothyroidism, diabetes mellitus, and other chronic cardiovascular, endocrinologic, or peripheral vascular conditions. This was confirmed through laboratory blood tests.

**Figure 1:**
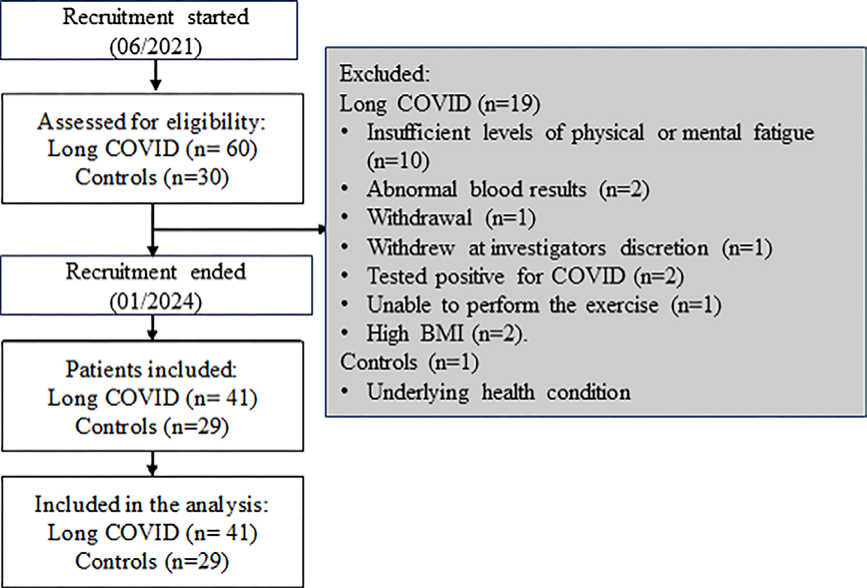
Flowchart shows inclusion and exclusion of participants with post–COVID-19 condition, commonly called long COVID, and healthy control participants. BMI = body mass index.

The control group consisted of healthy volunteers closely resembling the PCC cohort by exhibiting low to moderate physical activity. Individuals in the control group were required to have been free from COVID-19 infection for 3 months prior to enrollment, with no symptoms of fatigue or fatigue causing conditions; this was confirmed verbally. No further blood investigation was clinically warranted. Additionally, individuals with contraindication to MRI were excluded. A flowchart of study inclusion and exclusion is provided in Figure 1.

### Procedures

Demographics and COVID-19 history were recorded for all participants. Participants with PCC underwent several screening tools to ensure there were no other underlying causes of fatigue, including clinical examination to measure blood pressure, heart rate, oxygen saturation levels, and body mass index (BMI) as calculated from height and weight. As a screening tool, blood samples were taken from all participants with PCC to assess levels of glycated hemoglobin, B-type natriuretic peptide, N-terminal pro–brain natriuretic peptide, creatinine, estimated glomerular filtration rate, hemoglobin, alanine transaminase, and bilirubin to ensure they were within reference ranges (Appendix S1). The CFQ-11 with Likert scoring system was used to quantify both physical fatigue (items 1–7) and mental fatigue (items 8–11). The Likert scoring system involves questions answered from a 4-point scale ranging from asymptomatic to maximally symptomatic, with a total score calculated out of 33. Fatigue ranges within a score of 0–9 for normal, 10–15 for mild, and greater than 16 for moderate to severe. Transthoracic echocardiography was also performed for screening purposes to confirm normal cardiac structure and function. Physical examination, blood testing, and echocardiography were performed to exclude any other potential cause of fatigue; this was determined by a specialist cardiologist who had 10 years of experience.

**Figure 2:**
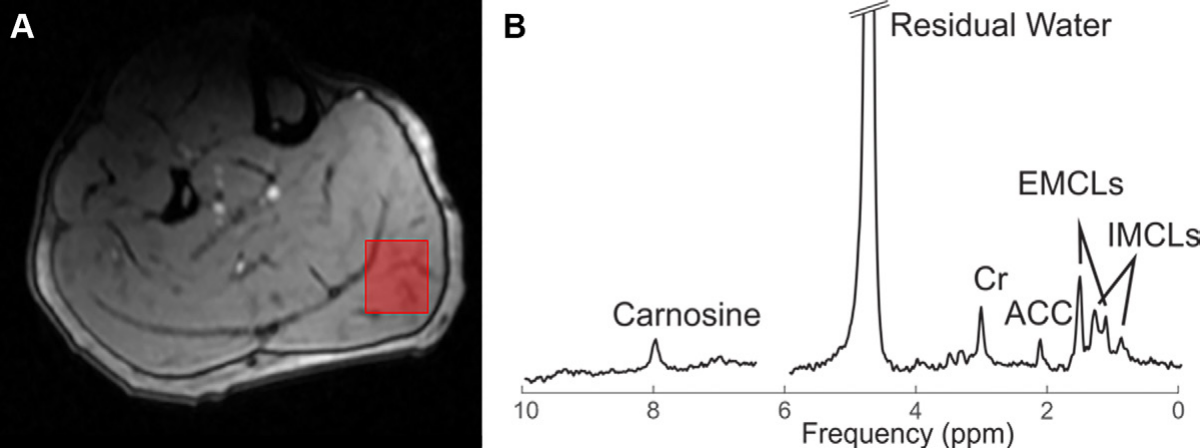
**(A)** Noncontrast MRI scan during ^1^H MR spectroscopy (resting assessment) in a 32-year-old healthy male control participant shows the voxel of interest (red box) placed on the target muscle (gastrocnemius medialis) while avoiding surrounding muscles. **(B)** Graph shows proton spectral data during rest. ACC = acetylcarnitine, Cr = creatine, EMCLs = extramyocellular lipids, IMCLs = intramyocellular lipids.

***MR spectroscopy protocol.—***All participants underwent ^1^H and dynamic ^31^P MR spectroscopy of the gastrocnemius muscle using a whole-body 3-T MRI system (MAGNETOM Prisma; Siemens Healthineers). An exercise band (40–80-lb [18–36-kg] exercise band; TOMSHOO) was secured to the bottom of the dominant foot. A dual-tuned ^1^H or ^31^P coil (Rapid Biomedical) was secured around the calf with straps. For ^1^H MR spectroscopic localization, a stimulated-echo acquisition mode sequence was used, with the voxel of interest (20 × 20 × 20 mm^3^) positioned on the gastrocnemius medialis while avoiding subcutaneous fat tissue (Fig 2).

**Figure 3:**
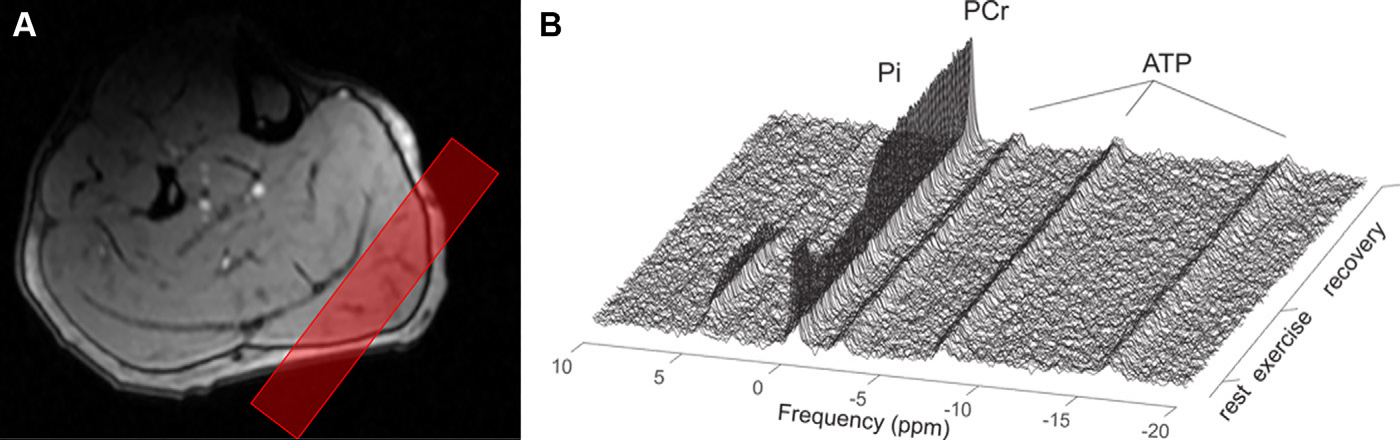
**(A)** Noncontrast MRI scan during ^31^P MR spectroscopy (dynamic assessment) in a 32-year-old healthy male control participant shows the voxel of interest (red box) placed on the target muscle (gastrocnemius medialis) while avoiding adjacent muscle groups. **(B)** Graph shows dynamic spectral data during rest, exercise, and recovery whereby peaks correspond to inorganic phosphate (Pi), phosphocreatine (PCr), and adenosine triphosphate (ATP).

Dynamic ^31^P MR spectroscopy was performed using a depth-resolved surface coil spectroscopic acquisition sequence (20,21). A 20-mm slab was positioned obliquely through the gastrocnemius medialis muscle while avoiding other muscle groups (Fig 3). Participants were asked to rest for 1 minute followed by 2–5 minutes of exercise. Exercise time depended on the ability of each participant to maintain exercise intensity, with a minimum exercise time of 2 minutes based on previous studies that showed a new metabolic steady state is reached 2 minutes after the onset of exercise (21,22). Individuals were asked to stop exercising if their effort decreased, and their total exercise time was recorded (2.5 minutes [*n* = 2], 3 minutes [*n* = 44], 3.5 minutes [*n* = 1], 4 minutes [*n* = 18], 5 minutes [*n* = 5]). More details are provided in Appendix S1.

### Statistical Analysis

Spectra were analyzed using the Oxford Spectroscopy Analysis fitting toolbox (23), with MATLAB (version R2021b; MathWorks) implementation of the advanced method for accurate, robust, and efficient spectral fitting (AMARES) (24).

All evaluated parameters were summarized using descriptive statistics, including means and CIs. Means and SDs for each group are provided, and differences between groups were calculated using the *t* test. Quantile-quantile plots were used to test for normality in the data. SPSS (version 29.0.1.0; IBM) was used to perform the statistical analysis. General linear models using the *F* test were used to assess differences between the two groups for all MR spectroscopic parameters, including corrections for age, sex, and BMI. Linear mixed models with Wald *z* statistics were used to assess dynamic differences (phosphocreatine, ADP, pH) between groups (participants with PCC, controls) from rest, end of exercise, and end of recovery. Linear mixed models were also corrected for age, sex, and BMI. Random effects included all participants. Results included the main effects of group, time, and group multiplied by time interactions (hereafter, group*time). Time was modeled as discrete time points (rest, end of exercise, end of recovery). Pearson correlations were used to assess relationships between the total CFQ-11 Likert score and MR spectroscopic parameters, and then between age and all MR spectroscopic parameters in participants with PCC. *P* < .05 was considered indicative of a statistically significant difference. The data acquisition and subsequent analysis (Appendix S1) (25–29) were performed by a postdoctoral researcher (L.E.M.F.) who participated in recruitment and acquisition of the MRI; however, the quality control process was blinded and performed by an author (L.V.) and verified by a second author (B.R.).

## Results

### Participant Characteristics

A total of 60 participants with PCC and 30 healthy control participants were screened. Of these, 41 participants with PCC (mean age, 44 years ± 9 [SD]; 23 male, 18 female) (mean BMI, 26 ± 4) and 29 healthy control participants (mean age, 34 years ± 11; 18 male, 11 female) (mean BMI, 23 ± 3) were included in the study. Patients with PCC had a moderate to severe mean fatigue score of 29 ± 3 using the Likert scale. All control participants were healthy and reported no symptoms of fatigue. Details are provided in Table 1. All screening blood test parameters were within reference ranges for participants with PCC who were included in the study.

**Table 1:**
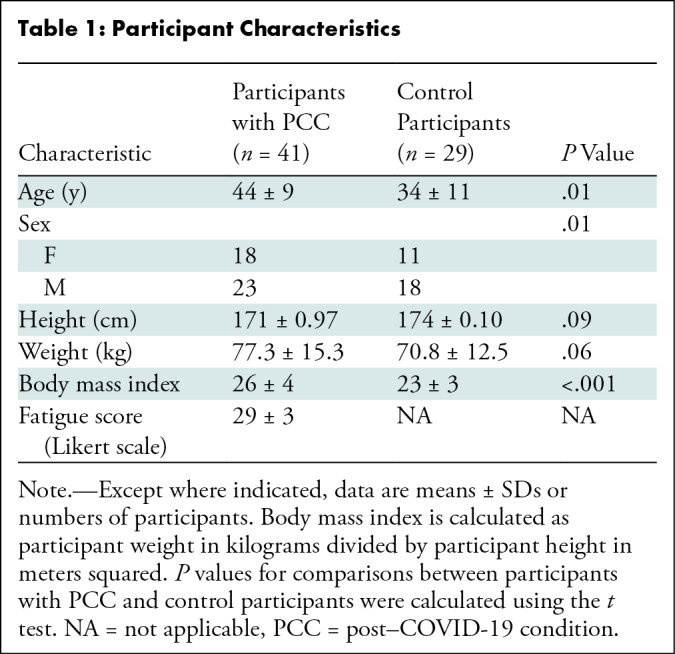
Participant Characteristics

### MR Spectroscopic Analysis

Proton spectroscopic results showed participants with PCC and controls had comparable intramyocellular lipid content (mean difference, 0.18; 95% CI: 0.16, 0.24; *P* = .60) (Table 2). There was also no evidence of a difference in creatine (mean difference, 8.21; 95% CI: 24.9, 41.3; *P* = .34) or acetylcarnitine (mean difference, 4.28; 95% CI: 2.6, 11.2; *P* = .12) levels between the two groups. On the other hand, the carnosine level was significantly higher in the control group, with a mean difference of 1.15 mmol/L (95% CI: 0.50, 1.88; *P* = .007).

**Table 2:**
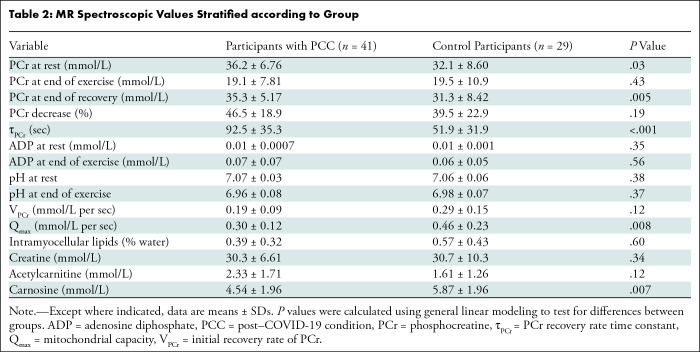
MR Spectroscopic Values Stratified according to Group

Phosphorus spectroscopic results showed participants with PCC had higher phosphocreatine levels at rest relative to controls (mean difference, 4.10 mmol/L; 95% CI: 0.77, 8.16; *P* = .03), but there was no evidence of a difference in phosphocreatine levels at the end of exercise between groups (mean difference, 0.40 mmol/L; 95% CI: 2.96, 10.9; *P* = .43). The percentage decrease in phosphocreatine during exercise was also comparable between the PCC and control groups (46.5% vs 39.5%, respectively; *P* = .19). Values for each group are summarized in Table 2. Linear mixed models were used to assess dynamic differences in phosphocreatine over the three time points (rest, end of exercise, end of recovery) (Fig S1). From rest to the end of exercise (including both groups), phosphocreatine levels decreased by a mean of 13.7 mmol/L (95% CI: 8.74, 18.7; *P ≤* .001). There was then, from the end of exercise to recovery, a significant increase by a mean of 12.5 mmol/L (95% CI: 7.78, 17.3; *P ≤* .001). The mean change in phosphocreatine throughout each time point was not different between the two groups (mean difference, 2.65; 95% CI: 1.12, 6.41; *P* = .17). There was no interaction between group*time (*P* = .89).

There was no evidence of a difference in ADP values at rest (mean difference, 0.0005; 95% CI: 0.00003, 0.0009; *P* = .35) and at the end of exercise (mean difference, 0.003, 95% CI: 0.008, 0.07; *P* = .56) between the two groups. Using linear mixed models to assess dynamic changes in ADP over time, ADP changed from rest to the end of exercise (in both groups), but there was no evidence of a difference between the two groups over time (mean difference, 0.02; 95% CI: 0.01, 0.05; *P* = 0.22) (Fig S2). There was no interaction between group*time (*P* = .70). There was no evidence of a difference in pH levels at rest between the two groups (mean difference, 0.025; 95% CI: 0.001, 0.05; *P* = .38) and at the end of exercise (mean difference, 0.043; 95% CI: 0.003, 0.09; *P* = .07). Using linear mixed models to assess dynamic changes over time, pH (including both groups) changed from rest to the end of exercise, with a mean increase of 0.05 (95% CI: 0.02, 0.08; *P* ≤ .001) (Fig S3). There was no evidence of a difference in pH level over the two time points between groups (mean difference, 0.086; 95% CI: 0.06, 0.12; *P* = 0.78). There was no interaction between group*time (*P* = .92). The individual and mean changes in phosphocreatine, ADP, and pH levels across the three time points are depicted in Figures S1–S3.

**Figure 4:**
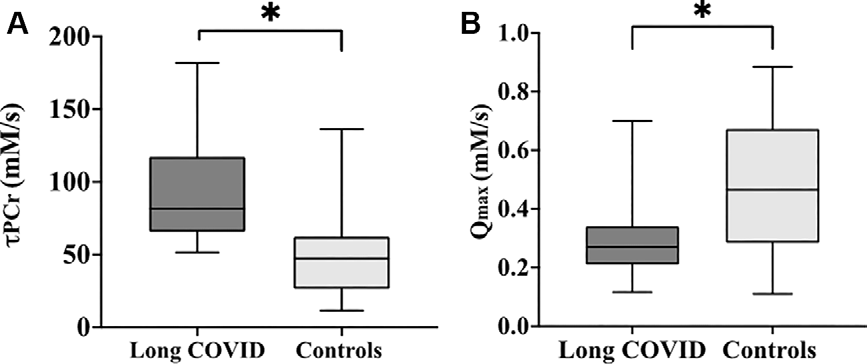
Box plots show the **(A)** mean phosphocreatine recovery rate time constant (τ_PCr_) and **(B)** mean mitochondrial capacity (Q_max_) (in millimolars per second; 1 mM = 1 mmol/L) between participants with post–COVID-19 condition, or long COVID, and control participants. The error bars represent SDs. * denotes a statistically significant difference (*P* ≤ .05).

**Figure 5:**
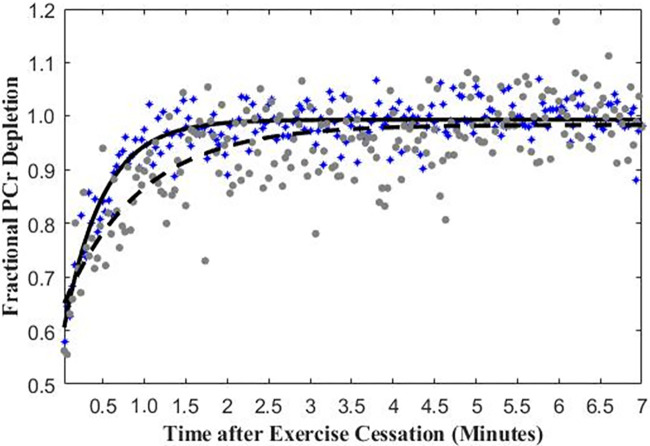
Graph shows phosphocreatine (PCr) recovery following exercise cessation in a participant with post–COVID-19 condition (PCC, or long COVID) (gray) and a control participant (blue). Black lines (dashed line for the participant with PCC, solid line for the healthy control participant) represent the exponential fitting of the phosphocreatine recovery rate time constant.

Participants with PCC had a longer mean τ_PCr_ (92.5 seconds ± 35.3) compared with controls (51.9 seconds ± 31.8) (mean difference, 40.6; 95% CI: 24.3, 56.6; *P* ≤ .001) (Fig 4). Phosphocreatine recovery in a participant with PCC and a control participant is depicted in Figure 5. Q_max_ was higher in the control group (Fig 4), with a mean difference of 0.16 mmol/L per second (95% CI: 0.07, 0.26; *P* = .008) between groups.

There was no evidence of a relationship between fatigue scores and phosphocreatine at rest, pH at rest, ADP at rest, phosphocreatine at the end of recovery, phosphocreatine decrease, τ_PCr_, pH at the end of exercise, ADP at the end of exercise, initial recovery rate of phosphocreatine, Q_max_, creatine, intramyocellular lipids, acetylcarnitine, and carnosine (r ≤ 0.25, *P* ≥ .10). There was also no evidence of a relationship between participant age and any of the abovementioned MR spectroscopic parameters (r ≤ 0.25 and *P* ≥ .10 for all variables).

## Discussion

The cause of fatigue in individuals with post–COVID-19 condition (PCC), commonly called long COVID, remains speculative as the condition is not yet fully understood. To our knowledge, this is the first study to quantify metrics using multinuclear MR spectroscopy that are implicated in mitochondrial dysfunction in participants with PCC relative to healthy controls. Both the MRI and exercise protocols were well tolerated among all participants, with no dropouts. The study findings indicate significant differences between participants with PCC and controls in terms of MR spectroscopic parameters linked to mitochondrial function and adenosine triphosphate (ATP) resynthesis, such as carnosine, phosphocreatine recovery time constant, and mitochondrial capacity. However, there was no relationship between MR spectroscopic parameters and fatigue scores.

Because of the predominant fatigue symptoms, a link between PCC and myalgic encephalomyelitis–chronic fatigue syndrome (ME/CFS) has been proposed (6). The pathophysiologic features of ME/CFS appear to involve multiple systems (30), with mitochondrial dysfunction considered a potentially key factor (31). This is further supported by muscle biopsy studies demonstrating oxidative damage, impaired oxidative phosphorylation, and lower ATP production in participants with ME/CFS (32–34). The current study demonstrated that, like ME/CFS, participants with PCC have signs of mitochondrial dysfunction, evidenced by a prolonged mean τ_PCr_ of 92.5 seconds ± 35.3 (SD) compared with 51.9 seconds ± 31.9 in controls. Notably, previous studies suggested that other patient groups experiencing breathlessness and fatigue, such as those with heart failure, may also show prolonged τ_PCr_, suggestive of reduced oxidative capacity following exercise (35). The control values are in good agreement with previous research studies that found a mean τ_PCr_ of 44.4 seconds ± 18 following plantar flexion exercise with a constant workload (21). The difference in τ_PCr_ between healthy and diseased states holds clinical and scientific importance as it potentially serves as an in vivo marker of mitochondrial dysfunction in PCC therapeutic trials. It also highlights the ATP-phosphocreatine system may take longer to replenish phosphocreatine and ATP after exercise, impacting energy production and pointing toward inefficiencies in pathways, including cellular respiration, ATP synthesis, and substrate utilization.

The current study also found significant differences in other parameters indicative of mitochondrial oxidative capacity, such as Q_max_, which was lower in the PCC cohort. This is consistent with recent data supporting the repression of mitochondrial oxidation genes and its downstream impact on the fatty acid oxidation pathway, which could be downregulated (9). Such changes can lead to mitochondrial dysfunction due to an inability to oxidize metabolic fuels affecting ATP levels, mitochondrial bioenergetics, and respiration (36).

Another important aspect of fatigue and muscle metabolism is pH. Previous MR spectroscopic research in ME/CFS has noted that patients with fatigue may have a higher pH level at rest and after recovery following exercise compared with healthy controls (37). In the current study there were minimal differences in resting pH and end-of-exercise pH levels between the two groups. This discrepancy may be due to variations in exercise intensity and duration and cohort size across studies. Further investigation using larger cohorts with standardized exercise protocols are needed to infer the role of pH in PCC fatigue. Alternatively, changes in mitochondrial function in PCC may differ from ME/CFS, with a stable pH level possibly being a distinctive feature of PCC. Despite a lack of difference in pH, we did find a difference in carnosine, with lower levels in the participants with PCC. In a healthy population, carnosine buffers high amounts of protons during glycolytic activity, which is useful in maintaining pH (38). A possible explanation for the lack of synergy between carnosine and pH results is that there may be some degree of compensatory buffering of protons in the PCC cohort, which is in keeping with subclinical metabolic acidosis in this group.

There were no differences in proton parameters, including the creatine level, and no difference in the changes of phosphocreatine levels before and after exercise. This supports the validity of previous assumptions of a constant phosphocreatine to total creatine level throughout measurements in the equation (no. 5) provided in Appendix S1 (28). Importantly, no group differences were found for acetylcarnitine levels, which is synthesized in the muscle from carnitine and acetyl‐CoA when mitochondrial acetyl‐CoA exceeds its usage by the tricarboxylic acid cycle. Thus, acetylcarnitine typically increases during energy production from fatty acids (ie, β-oxidation) (26). The lack of difference in muscle acetylcarnitine levels could indicate good usage of fatty acids as an energy source in participants with PCC.

It is also worth noting that there were no correlations between MR spectroscopic parameters and fatigue scores in participants with PCC. This may be due to the heterogeneity of the PCC population with differing types of symptoms, severity, and underlying mechanisms. The CFQ-11 questionnaire is also a self-reported qualitative measure that relies on individual interpretation of the questions and thresholds for reporting fatigue, limiting its reliability. Previous studies suggest the CFQ-11 may be more useful when studying intraindividual (longitudinal) responses to disease or treatment rather than cross-sectional effects. Future research should consider longitudinal assessments, potentially from the acute infection phase, and should incorporate pre- and postinfection physical activity questionnaires, T2 relaxometry, and fat fraction measurements at MRI to provide insights into muscle properties and oxidation. Overall, the differences found between the two groups in the current study imply that mitochondrial dysfunction may contribute to fatigue in PCC, offering a potential pathophysiologic explanation. Moreover, the study highlights that MR spectroscopy is a useful tool for monitoring potential mitochondrial function improvements during new treatment testing in clinical trials.

The main limitation of this study was the use of an exercise band for the dynamic protocol, making it difficult to standardize or quantify total workload during exercise. To counteract this, a minimum decrease in phosphocreatine of 20% (actual mean decrease of 46.5% and 39.5% in the PCC and control groups, respectively) was required to ensure an appropriate exercise intensity to elicit sufficient changes in phosphocreatine signal (39). Second, the exercise band has the potential to reduce skeletal muscle blood flow and oxygen supply, which may have affected metabolic measures. However, this seems unlikely, as differences in ischemic burden would cause differences in pH levels, which was not observed. Third, the variation in exercise duration across groups could be a limitation, but because phosphocreatine depletion reaches a steady state after 2 minutes of mild exercise, this is unlikely to affect results. Fourth, while the sample size was relatively small compared with larger population studies, it is considerable for an observational study in MR spectroscopy. Fifth, the mean age of the control group was slightly younger than that of the PCC group; therefore, the healthy controls may have a higher exercise capacity. However, previous reports showed little to no difference in dynamic ^31^P MR spectroscopic parameters between young and older populations (38,40), and the difference in parameters between groups persisted when adjusting for differences in age. Lastly, while great care was taken to ensure the participants with PCC had no other known fatigue cause, the study design does not allow for a direct comparison of a participant’s condition prior to COVID-19 infection. Therefore, we cannot exclude that the participants with PCC may have had a subclinical mitochondrial dysfunction before infection, with COVID-19 acting as a stressor on the system, potentially contributing to aberrant recovery.

Overall, this study found that participants with post–COVID-19 condition (PCC), or long COVID, exhibited differences in parameters suggestive of mitochondrial dysfunction and adenosine triphosphate resynthesis perturbations, as indicted by a prolonged phosphocreatine recovery rate time constant and mitochondrial capacity, when compared with controls. As a relatively new medical condition, these findings provide a better understanding of the pathophysiologic mechanisms of PCC. They also provide quantifiable in vivo measurement of mitochondrial function in a general patient population, offering further insights into the biologic basis of other fatigue-based conditions.
